# Functional Significance of Probiotic Bacterial Interactions with Milk Fat Globules in a Human Host

**DOI:** 10.3390/microorganisms13020223

**Published:** 2025-01-21

**Authors:** Withanage Prasadini Wasana, Mark Waterland, David W. Everett, Caroline Thum

**Affiliations:** 1 Food Function and Physiology Team, AgResearch, Palmerston North 4410, New Zealand; wasana.withanage@agresearch.co.nz; 2 Riddet Institute, Massey University, Palmerston North 4410, New Zealand; d.everett@massey.ac.nz; 3 School of Food Technology and Natural Sciences, Massey University, Palmerston North 4410, New Zealand; m.waterland@massey.ac.nz

**Keywords:** milk fat globules, probiotics, bacterial adhesion, interactions, host health

## Abstract

Dairy products often serve as matrices for delivering probiotic bacteria to humans through the diet; however, little is known about the impact of milk fat globules on the growth and survival of probiotic microorganisms. This review discusses current knowledge on the structure and functionality of the milk fat globule membrane (MFGM) and the structural components contributing to the mechanisms of interactions with probiotic bacteria. We analyzed studies published between 2001 and 2025 with reference to earlier foundational research on probiotics and MFGM structure to explore the functional significance of MFGM–probiotic interactions. Recent research indicates that the effects of MFGM interaction with bacteria are species-specific and may influence probiotic activity in the host, including enhancing probiotic viability during intestinal transit and modulating probiotic colonization. In general, research findings suggest that the MFGM holds potential for use as a probiotic carrier to the gut with beneficial health consequences.

## 1. Introduction

The human gastrointestinal (GI) tract harbors a complex and diverse population of microorganisms [1,2,3]. The collective assembly of these microorganisms residing and colonizing the GI tract, including bacteria, archaea, and eukarya, is known as the gut microbiota [1,4,5]. This microbial ecosystem has co-evolved with the host to contribute to a number of essential functions in the host through physiological functions such as strengthening gut integrity and modifying the intestinal epithelium, harvesting energy from digested food, protecting against pathogens, and regulating host immunity [3,4,6,7]. The microbial community in the GI tract undergoes several changes over the human lifespan from infant to adulthood. Host-specific and nonspecific factors, including age, diet, hygiene factors, and antibiotic exposures, affect the diversity of the gut microbiome [5,8,9].

Dietary composition has been identified as a critical determinant influencing the structural and functional attributes of the gut microbiota [8]. Milk, in particular, has been recognized as one of the most fundamental dietary components and plays a pivotal role in shaping gut microbial diversity and maintaining desirable morphological and biosynthetic properties [10]. These effects are observed both qualitatively and quantitatively and are sustained from generation to generation [10,11]. Milk provides valuable nutrients and immune components for the optimal growth of infants [12,13,14]. The fat portion of milk, mainly triglycerides within fat globules, is stabilized by a phospholipid tri-layer milk fat globule membrane (MFGM). The MFGM also contains cholesterol, bioactive lipids, and membrane-specific proteins [15,16]. Most fat globules are within the size range of 1 to 10 µm with an average of 3–4 µm in bovine milk as measured using a static light scattering technique [17], although other methods such as dynamic light scattering [17,18] are capable of showing sizes below 1 µm.

Milk fat globules (MFGs) and membrane components have a significant role in human health, including gut function, immune boosting, and neonatal growth and development [13,14,19,20]. MFGM components alter the gut microbiota and probiotic functionality by modulating bioactivity and the adhesion of the microbiome to the intestinal epithelium [11,21,22]. However, the mechanisms of how the MFGM interacts and modulates gut microbiota composition, metabolism, and, consequently, health outcomes are not fully understood [10,14,23,24].

This review discusses the current understanding of the structure and functionality of the MFGM, as well as the structural components involved in interactions with probiotic bacteria. The functional significance of these MFGM–bacteria interactions, particularly in influencing bacterial adherence to intestinal epithelial cells, altering bacterial metabolism, and the subsequent effects on the human host, is critically evaluated.

## 2. Methodology

To ensure a comprehensive review, studies on MFG, probiotics, and their interactions, published between 2001 and 2025, were selected and critiqued. Foundational research on probiotics and the structure of MFGM (published between 1943 and 2025) was also included to investigate the mechanisms underlying MFGM–probiotic interactions. Literature searches were carried out using PubMed, Scopus, ScienceDirect, SpringerLink, and Google Scholar databases, with keywords including “milk fat globule membrane”, “probiotics”, “MFGM-probiotic interactions”, “probiotic adhesion”, “intestinal colonization”, and “host health”. Only English-language studies were considered. This methodology ensured that the review captured the most relevant and current insights into the topic.

## 3. Milk Fat Globule Membrane Constituents and Functionality

MFGs are formed from the secretory cells of the mammary gland [14,25]. The synthesis of triglycerides starts inside or on the surface of the rough endoplasmic reticulum membrane and accumulates as microlipid droplets (MLDs) in the cytoplasm. Upon release into the cytoplasm, MLDs grow in volume by fusion with each other to form cytoplasmic lipid droplets (CLDs) of various sizes. The peripheral layer of the MFGM is formed during the excretion of the CLDs out of the epithelial cells by a process called milk fat globule “budding”. CLDs are encapsulated by the cell membrane before being expelled into the alveolar lumen. Upon closure of the cell membrane, some components of the cytoplasm are entrenched between the inner and the peripheral membrane layer, forming “crescents” on the inner side of the globule membrane [24,26,27].

The outer MFGM membrane is a bilayer of polar lipids with embedded glycoproteins, enzymes, and phosphoproteins [28,29]. The phospholipids are the main component of the polar lipid fraction. The major phospholipid species include phosphatidylcholine (PC, 14–38%), phosphatidylethanolamine (PE, 6–36%), and sphingomyelin (SM, 27–43%), with phosphatidylserine (PS) and phosphatidylinositol (PI) as comparatively minor components [19,25]. The primary lipid components of the bilayer are glycolipids, cerebrosides, gangliosides, and cholesterol, whereas the interior monolayer is mainly composed of PE, PI, and PS [24]. Liquid-ordered domains, sometimes referred to as lipid rafts in cellular bilayers, are typically formed as part of the outer MFGM bilayer by the phase separation of SM and cholesterol complexes [28,30,31]. Because of the densely packed structure and higher melting point, SM is essential for maintaining stability and appropriate fluidity of the membrane structure [30]. The lipid fraction of the MFGM is reported to confer numerous health benefits to the host, including regulation of cell growth, brain development, intestinal health and infant intestinal maturation, cognitive development, cholesterol distribution and homeostasis, and lipid metabolism [24,32,33,34].

MFGM proteins represent 25–60% of the total MFGM and comprise more than a hundred different proteins of 30–240 kDa molecular weight [35]. These proteins are dispersed unevenly within the MFGM in glycosylated and non-glycosylated forms [36]. Mucin 1 (MUC 1), xanthine oxidase (XO), lactadherin (PAS6/7), cluster of differentiation (CD) 36, butyrophilin (BTN), adipophilin (ADPH), and fatty-acid-binding proteins (FABPs) are the most abundant proteins in the MFGM [37,38,39,40]. The functional properties of these proteins include antiviral and antibacterial activity, lipid metabolism, intercellular communication, and the ability to prevent the binding of pathogens to intestinal cells. Toll-like receptors (TLRs), such as TLR2, TLR3, and TLR5, have been identified in milk and are considered to be among the minor proteins associated with the MFGM. The association of these TLRs with the MFGM is important for the rapid and effective recognition of bacterial cell wall components during the innate immune response [14,41,42].

Most of the carbohydrates in the MFGM are found as glycoconjugates, which include glycoproteins and glycolipids. Acid glycolipids (gangliosides) and neutral glycolipids are the two categories of glycolipids. GD3, GM3, and GT3 comprise 63–83% of gangliosides found in milk [43]. Gangliosides are made up of different residues connected by glycosidic bonds and oligosaccharides connected by ceramide to one or more sialic acids and are involved in neuronal development and immunological adaptations in neonates [11,25,44,45]. Neutral glycolipids, such as galactosylceramides, glucoceramides, and lactosylceramides, comprise one or more carbohydrate residues linked to ceramide in the lipid portion. Mucin, BTN, fatty acid transporter (CD 36), milk lectin PAS 6/7, and mucin are examples of glycoproteins present in the MFGM. Glycoproteins are important for preventing bacterial and viral infection and maintaining intercellular communication [45,46]. Table 1 provides a summary of experimental evidence demonstrating the health benefits of MFGM supplementation on human health, in particular, the effect on human health including growth and development, gut health, immunity, and cognitive development.

Although the health benefits of MFGM supplementation, such as improvements in growth and development, gut health, immunity, and cognitive development, are well documented [24,32,34], there are conflicting findings and limitations in the current body of research. For instance, variations in study outcomes can be attributed to differences in the source of MFGM, processing methods, or bioavailability in specific populations. Some studies, particularly those using animal models, may not fully translate to human physiology, and human trials are often limited by small sample sizes or short durations [43,44].

## 4. Benefits of Probiotic Bacteria

The gut microbiome, essential for human health, is a diverse and intricate community. The GI tract contains approximately 10^13^ to 10^14^ microorganisms [63], with about ten times the number of bacterial cells compared to human cells and over one hundred times the genomic content of the human genome [2,4,64]. Due to the influence on host metabolism, physiology, nutrition, and immunological function, this community is often described as our “secret metabolic organ” [3].

Probiotics are defined as “Live microorganisms which, when administered in adequate amounts, confer a health benefit on the host” by the World Health Organization and the Food and Agriculture Organization of the United Nations [65]. Probiotic organisms, which predominantly exist in the large intestine, can play a beneficial role in the host by preserving the equilibrium of gut microorganisms, with some also exerting effects in the small intestine. The lactic acid bacteria (LAB) group includes most known probiotic species, such as *L. acidophilus*, *L. rhamnosus*, *L. fermentum*, *L. casei*, *L. lactis*, *L. delbrueckii* subsp. *bulgaricus*, *L. paracasei*, *L. plantarum, L. reuteri*, *L. gasseri*, *L. helveticus*, *L. johnsonii*, and *L. sporogenes.* Additional probiotic strains that hold potential for commercialization are Bifidobacterium species, such as *B. lactis*, *B. animalis*, *B. breve*, *B. longum*, *B. infantis*, and *B. adolescentis* [7,9,66,67]. Characteristics such as resistance to bile and gastric acids; adherence to mucus, human epithelial cells, and cell lines; antimicrobial activity against potentially pathogenic bacteria; capacity to decrease pathogenic adhesion to surfaces; bile acid hydrolase activity; stimulation of mucin production; and modulation of the gut-associated lymphoid tissue are necessary for these probiotic bacteria to function effectively [7,68,69].

In recent years, research into probiotics has advanced significantly, as reported by numerous studies demonstrating the critical function that probiotics play in supporting immunity and preserving human health. The most prominent activities of probiotics are immune system modulation, epithelial barrier maintenance, and pathogen adhesion prevention on the gut surface [3,70,71]. These activities may also be useful in managing long-term inflammatory conditions, such as Crohn’s disease, with additional anti-diabetic, anti-carcinogenic, and anti-obesity effects as demonstrated in clinical trials [7,72,73]. Probiotics greatly regulate the intestinal microbiota [7,67]. For example, Bifidobacteria are used to treat and prevent intestinal disorders due to their ability to colonize the intestinal lining [74]. Bifidobacteria release adhesins that can cling to intestinal epithelial cell receptor proteins and compete with pathogens for the same receptor to prevent pathogenic microbes from growing and colonizing [75]. Piewngam and colleagues reported that oral intake of probiotic *Bacillus subtilis* spores could inhibit *E. faecalis* translocation from the gut to the bloodstream and subsequent systemic infection in mice by inhibiting *E. faecalis* FSr (fecal streptococci regulator) quorum sensing activity [76]. According to Fan et al., the “next-generation” probiotic strain, *Bacteroides fragilis* ZY-312, enhances the relative abundance of Bacteroides and Bacillus in neonatal rats, restores the function of the intestinal epithelial barrier, and prevents necrotizing enterocolitis caused by *Cronobacter sakazakii* [77]. To preserve homeostasis in the immediate environment, lactobacilli may interact with commensal bacteria in the gut to lessen the colonization and proliferation of multidrug-resistant Enterobacteriaceae [78].

Probiotics are also important in maintaining a well-balanced gut microbial population [72]. This is important for both the host and the microbiota to coexist in a mutually beneficial relationship as disrupting this homeostasis can result in gut microbial imbalance, known as dysbiosis. This term refers to an altered composition of the gut microbiota and can manifest in various forms including reduced microbial diversity, overgrowth of pathogenic microbes, or an imbalance between beneficial and harmful microbes. These disruptions can impair the ability of microbiota to maintain host health and wellness. This may further lead to metabolic and GI disorders including obesity, diabetes, inflammatory bowel disease, Crohn’s disease, ulcerative colitis, colorectal cancer, and antibiotic-associated diarrhea [2,4,66,72]. Table 2 presents clinical evidence highlighting the main health benefits of probiotic supplementation, summarizing the effects of probiotics on gut microbiota, gut health, and immune modulation, and demonstrating how probiotics help improve microbial diversity, support digestive health, and enhance immune function.

Probiotic research faces several limitations and challenges that impact the interpretation of findings. Many studies involve small sample sizes, lack long-term follow-up, or focus on specific populations, which restricts the generalizability of results. Additionally, inconsistencies in study designs, including variations in probiotic strains, dosages, and treatment durations, contribute to discrepancies in outcomes. The mechanisms of action for many probiotic strains remain unclear, and health benefits are highly strain-specific. Individual variability also plays a significant role, as factors such as age, genetics, gut microbiota composition, and dietary habits influence the effectiveness of probiotic interventions. These challenges highlight the need for personalized approaches to probiotic supplementation and further research to address gaps and inconsistencies.

## 5. Effects of Milk Fat Globule and Probiotic Interaction

### 5.1. Probiotic Growth and Survival

Probiotics are easily inactivated by factors such as pH, oxygen, temperature, and moisture, which can reduce or eliminate the potential to retain beneficial qualities after ingestion [9,74]. Probiotic efficacy and bioactivity are thought to be best when cells survive GI transit, yet probiotics have difficulties under the harsh chemical and physical conditions of the GI tract. The survival of probiotic bacteria in the GI tract depends on the ability to endure acidic conditions (stomach acidity pH 1–2), bile salts (bile salt concentration 0.3–0.5%), bile salt hydrolase activity, and the duration of exposure to these stressors [99].

MFGM may increase probiotic survival through stimulation of exopolysaccharide (EPS) production. EPSs are biological polymers secreted by microorganisms, including LAB and Bifidobacteria, to enable survival under severe environmental conditions. EPSs are one of the primary elements in creating an extracellular biofilm matrix that shields microorganisms from harmful conditions, such as extreme pH values and temperatures, antibiotics, and host immune defenses. Cell-surface-associated EPS from *Bifidobacterium breve* UCC2003 provided stress tolerance and promoted in vivo persistence of the bacteria [100]. Probiotic bacteria are also known to form biofilms, which are advantageous in encouraging colonization and prolonging persistence in the mucosa of the host while preventing colonization by harmful bacteria [101]. Zhang et al. reported that EPS production and biofilm formation by LGG were enhanced by bile stress and were further increased by MFGM-10 supplementation to increase the survival of LGG. However, in the absence of bile, MFGM-10 exposure reduced biofilm formation [16]. Raz et al. reported that small MFGs provide greater availability of nutrients for bacterial growth, whereas large MFGs have a bactericidal effect and induce biofilm formation. This study found that inhibition of biofilm formation in the presence of large MFGs can be achieved by adjusting the PE level to the concentration found in small MFGs [102].

Bile stress poses a significant obstacle for probiotics reaching the large intestine in a viable state. Gallier et al. studied the adsorption of bile salts to milk phospholipids and phospholipid–protein monolayers at the air–water interface under simulated intestinal conditions [103]. This study provides a basic understanding of the interfacial changes occurring at the surface of MFGs and milk phospholipid liposomes during passage through the duodenum. Their investigation revealed that adding bile to phospholipid and phospholipid–protein monolayers produced distinct structural characteristics, including branching and clustering of liquid-ordered domains and the potential creation of bile-salt-rich regions inside these domains [103]. This may avoid direct damage to probiotics once bound to the MFGM. The presence of a commercial MFGM preparation (MFGM-10, Arla Food Ingredients, Aarhus, Denmark) increased *Lacticaseibacillus rhamnosus* GG (LGG) survival against bile stress, which impacted bacterial survival under physiological conditions in the digestive tract [16]. In another study on the protection efficacy of MFGM on *Bifidobacterium longum* ssp. *infantis* ATCC 15697 against bile stress, 0.2% porcine bile salts reduced bacterial cell viability and damaged cell integrity [104]. Gene expression related to ATP-binding cassette transporter, galactose metabolism and transport, branched-chain amino acid transport, amino acid metabolism, pyruvate metabolism, and histidine metabolism was restored by the MFGM.

The MFGM provides a carbon source to support the growth and survival of probiotic bacteria in the colon, thus having a prebiotic effect. Bifidobacteria can produce extracellular glycosidases to digest glycans and glycolipids of the MFGM. *Bifidobacterium* spp. and *Lacticaseibacillus paracasei* isolated from dairy products can survive in carbohydrate-restricted media by using membrane-bound sugars on the MFGM [105].

The MFGM may also increase probiotic survival through encapsulation, a common technique for protecting bacteria and preserving viability and functionality throughout food processing and digestion. The natural composition of the MFGM may offer superior biocompatibility and functionality within dairy matrices, providing a more targeted and efficient encapsulation. Encapsulation by MFGM components primarily occurs via interactions between the hydrophobic lipid components of the MFGM and the bacterial cell membrane. By creating a barrier surrounding the probiotics, the glycoproteins and phospholipids in the MFGM can increase resistance to external stresses such as bile, pH, and digestive enzymes. As an example, isolated glycoprotein MUC1 effectively encapsulates LAB and protects bacterial cells during GI passage digestion with delivery to the appropriate site in the gut [35]. Furthermore, a recent study by Yadav et al. provides further evidence supporting this concept [106]. These authors show that human MFGM can encapsulate probiotics, ensuring safe passage through the gastrointestinal tract. This natural encapsulation mechanism not only protects probiotics from bile, low pH, and digestive enzymes but also enhances delivery to the gut, where beneficial effects are exerted. Furthermore, these authors emphasize the synergistic effects of human MFG with probiotics, such as reducing oxidative stress and inhibiting pathogenic growth. These findings reinforce the potential of MFGM-based encapsulation in functional foods and probiotic supplements to improve targeted delivery and enhance probiotic survival [106].

### 5.2. Probiotic Adhesion to the MFGM

**Figure 1:**
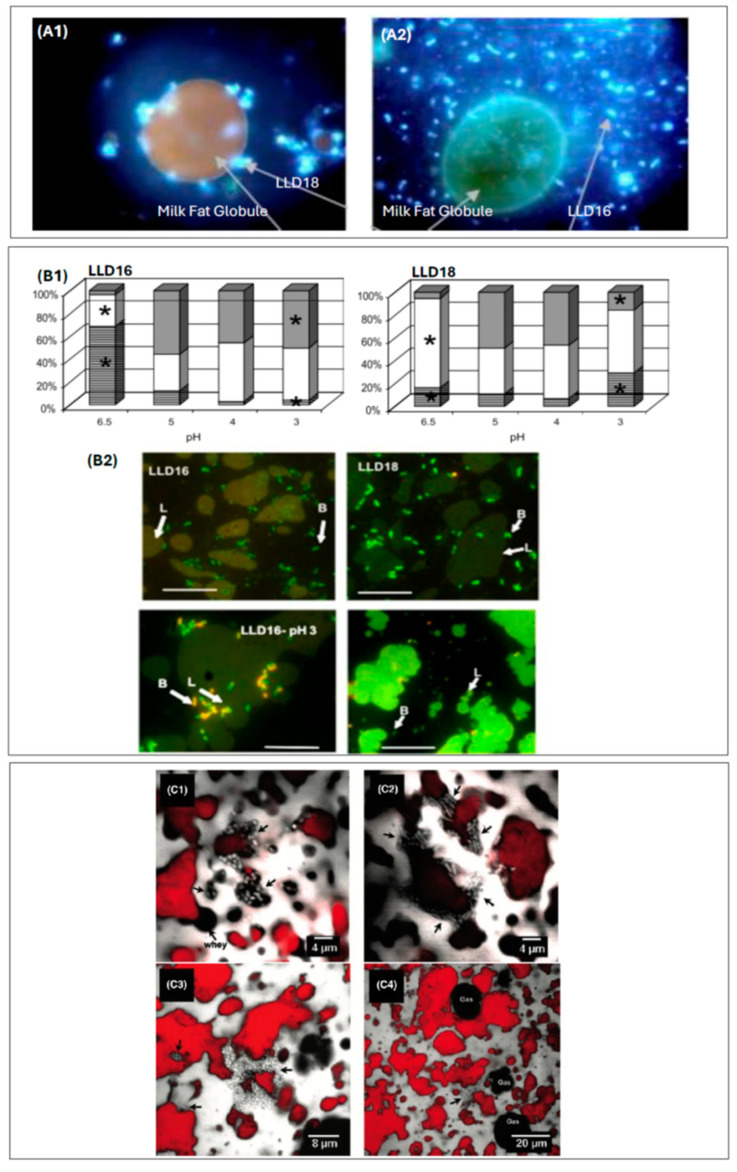
Association of probiotics with dairy matrices. (**A**) Images from fluorescence microscopy showing *Lactobacillus subtilis* subsp. *diacetylactis*: (**A1**) the LLD18 variant is clearly attached to the MFGM, whereas in (**A2**), the LLD16 variant is not [110]. (**B**) Localization of probiotics in a cream emulsion acidified to the indicated pH by lactic acid addition: (**B1**) percentage of cells, in gray inside the lipid phase, in white at the interface between lipid and serum, and in black horizontal lines in the serum phase, significant differences (*p* = 0.05) between strains are indicated by *; (**B2**) micrographs of cells added to the cream emulsion at pH 6.5 (**up**) or at pH 3 (**down**). L: lipid droplets, B: bacteria [111]. (**C**) Micrographs showing localization of bacteria in Emmental cheese. The protein network and bacteria are colored in gray, fat is colored in red, and whey pockets and gas microbubbles appear as black; (**C1**) at 1 day showing bacteria in a pocket of whey surrounding fat; (**C2**) after 12 days at 12 °C; (**C3**) after 8 days at 4 °C; (**C4**) at the end of ripening. Bacteria and/or colonies are indicated by arrows [109].

LAB and bifidobacteria in dairy food products have been shown to be located close to the MFGM and become part of the outer phospholipid bilayer or inside fat globules after initially being preferentially located near the fat/protein interface in cheeses [107,108,109,110,111]. Laloy et al. visualized the location of bacteria in cheddar cheese by transmission electron microscopy and reported that the microstructure and fat content of cheese affect the distribution and survival of bacteria [107]. A comparison of full-fat, reduced-fat, and free-fat cheddar cheese demonstrated the importance of fat in enhancing the retention of bacteria in the cheese matrix. After one and two months of ripening, the number of ghost cells, resulting from autolysis, increased, and bacteria appeared to be embedded in the MFGM or directly in contact with the fat–water interface. Bachiero et al. studied the quantitative affinity between different milk lipids and LAB using immunoblotting techniques and reported interactions with phospholipids, but no interactions with triacylglycerols [112]. Figure 1 illustrates the location of probiotics within different matrices, the impact of external factors such as pH, and dynamic interactions between bacteria and a cheese matrix during ripening.

Interactions between MFGs and probiotic organisms can promote probiotic adhesion to the intestinal epithelium through different mechanisms. MFGM- or milk phospholipid (MPL)-enriched media are nutrient sources for probiotics, providing energy and even changing the surface characteristics, such as increasing the ζ-potential at the surface of shear. Furthermore, probiotics positioned near fat–protein interfaces in dairy products may adhere to or be embedded within the MFGM, which strengthens adhesion to the intestinal lining [113]. Probiotic survival under gastrointestinal conditions and contact with intestinal epithelial cells can be improved by integration of probiotic cells into the outer phospholipid bilayer or by association with the fat–water interface [113,114], as summarized in Table 3. In these studies, increased adhesion of probiotics to intestinal cells was reported when grown in the media supplemented with MPL compared to adhesion without MPL.

In addition to increasing probiotic adherence, the MFGM can also prevent the colonization of harmful pathogens on the intestinal epithelium. Probiotics adhere to the intestinal epithelium more efficiently, reducing the available epithelial area for pathogens to become established. This effect has been demonstrated by the enhanced adhesion of *Lactobacillus* and *Bifidobacterium* when exposed to MFGM, leading to a significant decrease in pathogen colonization [55]. Although the adhesion of probiotics to the intestinal epithelium can be enhanced by the MFGM, not all strains show this behavior. For example, *L. rhamnosus* (LGG) was reported to reduce adhesion to the gut epithelium due to blocking of LGG pili adhesive sites by the MFGM [117], suggesting that interactions between MFGs and probiotics can be strain-specific.

### 5.3. Probiotic Gene Expression

MFGs can interact with probiotic bacteria to modulate gene expression, affecting adhesion to the intestinal epithelium and growth and survival in the gut. Factors such as bioactive lipids, membrane-associated proteins, and nutrient availability play a role in this process [10]. Changes in gene expression can increase or decrease the effectiveness of probiotic adhesion.

*Lactobacillus* and *Bifidobacterium* produce different surface proteins that play a crucial role in attachment to epithelial cells in the gut. These proteins include adhesins and sortase-dependent proteins, which are specifically designed to facilitate bacterial adherence. The expression of genes responsible for adhesion-related proteins can be enhanced by the interaction between MFG and probiotics. Rocha-Mendoza et al. reported increased gene expression of cell and mucus-binding protein A (CmbA), collagen-binding protein (Cnb), adhesion-like factor (EF-Tu), mucus adhesion-promoting protein (MapA), mucus-binding protein (MuB), and surface-layer protein (Slp) in *Pediococcus acidilactici*, *L. paracasei*, and *Lactobacillus reuteri* when cultured with milk phospholipids [114]. Although the study reported a clear correlation between milk phospholipids and enhanced bacterial adhesion, it did not fully explain the molecular mechanisms involved. The authors suggest that components of milk phospholipids may interact with bacterial cell membranes or signaling pathways, triggering transcriptional changes in adhesion-related genes.

MFG probiotic interactions can also enhance the expression of stress response genes in probiotics which protect against hostile conditions, such as the acidic environment of the stomach or bile salts in the intestine. The effect of MFGM on *L. rhamnosus* (LGG) revealed an increase in the survival of this probiotic organism under bile stress conditions by upregulating genes associated with EPS biosynthesis. These genes include the transcriptional regulator of polysaccharide biosynthesis (*wzr*), which is crucial for the regulation of EPS production, contributing to a protective barrier against bile salts. The genes galactophosphotransferase (*welE*) and glycosyltransferase (*welG*) are involved in the biosynthesis of the EPS matrix by transferring sugars to form the polysaccharide backbone, further enhancing bacterial protection under bile stress. Additionally, phosphotyrosine protein phosphatase (*wzb*) regulates the phosphorylation of proteins that modulate stress response pathways, contributing to bacterial survival by maintaining cellular integrity and function in hostile conditions. Together, these gene products contribute to stress resistance by facilitating EPS production and supporting bacterial cell protection under acidic and bile stress conditions [16]. A more recent study on *Bifidobacterium longum* subsp. *infantis* ATCC found that MFGM can effectively protect probiotic bacteria from bile stress [104]. In this study, the presence of MFGM was shown to restore the expression of genes associated with some metabolic pathways, including ATP-binding cassette (ABC) transporters, galactose metabolism and transport, branched-chain amino acid (BCAA) transport, and amino acid metabolism. These findings suggest that the MFGM plays a vital role in enhancing the resilience and functionality of probiotics by modulating gene expression related to adhesion, growth, and survival.

### 5.4. Host Health

Probiotics must be able to endure GI tract transit with retention of functionality, adhere to the intestinal epithelium, and proliferate in the colon to have health benefits [6,75]. These organisms do not permanently colonize the gut; certain strains may adhere to the intestinal lining for a short period and exert beneficial effects, such as modulating the gut microbiota and boosting immune response. Regular intake of probiotics is usually necessary to maintain these benefits.

Interactions between probiotics and MFGs can potentially affect the host in a number of ways, such as in digestion, immune modulation, and gut health. Probiotic interactions with MFGs can create a more favorable environment for probiotics to colonize the host gut as discussed in Section 5.2. These interactions may aid in balancing the gut microbiota, improving overall gut health, and potentially reducing the occurrence of gastrointestinal disorders such as diarrhea or irritable bowel syndrome [11].

MFG probiotic interactions may also improve the absorption of certain nutrients, including fat-soluble vitamins and fatty acids, in the host. Morifuji et al. observed the co-ingestion of sphingomyelin/MPL concentrate with fermented milk containing *Lactobacillus delbrueckii* subsp. *bulgaricus* 2038 and *Streptococcus thermophilus* 113 increased the absorption of dietary sphingomyelin in rats by approximately two-fold compared to sphingomyelin/MPL concentrate alone [115]. Probiotic bacteria can produce lipases that help digest fat globules more efficiently. This cooperative action between MFGs and probiotics increases the breakdown of sphingomyelin into components such as ceramide and phosphocholine which can then be absorbed by the host.

Probiotics are known to reduce NF-ĸB activation, thereby decreasing inflammation. A combination of MFGM and probiotics was reported to decrease NF-ĸB activation in a host compared to MFGM or probiotic treatments alone [12]. The MFGM is rich in bioactive lipids, such as sphingomyelin and phospholipids, which have known anti-inflammatory properties. When MFGM and probiotics are combined, the individual anti-inflammatory effects are amplified, resulting in a stronger anti-inflammatory response. Favre et al. reported that a combination of probiotics and MFGM had a higher mucosal B- and T-cell proliferative response in the host than either probiotic or MFGM treatments alone due to the synergistic effects on antigen presentation, immune modulation, and cytokine production leading to a more potent and efficient immune response [116].

Table 3 provides a summary of in vitro and in vivo studies exploring the effects of MFG and probiotic interactions, focusing on the impact on probiotic growth and survival, adhesion to the intestinal epithelium, and overall benefits for host health.

## 6. Mechanisms of Probiotic and MFGM Interaction

It is essential to understand the mechanisms by which bacterial cells bind to MFGM and MFGs to understand the impact of milk processing and digestion on growth, survival, and ultimately host health. Probiotic cells can generally adhere to the MFGM surface through physical or chemical interactions.

### 6.1. Physical Mechanisms

Bacterial adhesion to surfaces involves the initial diffusion of cells, followed by adsorption and attachment if the particle is sufficiently close to the surface [118]. Bacterial cells grow on surfaces in preference to the surrounding aqueous phase [119]. Physical forces, such as Brownian motion, van der Waals attraction forces, surface charge, and hydrophobic interactions, as well as chemotaxis and possibly haptotaxis, all contribute to the movement of bacteria toward or away from a surface [120,121,122].

Several models have been proposed to explain the effect of electrostatic forces that dictate interactions between bacterial and substrate surfaces, including the Derjaguin–Landau–Verwey–Overbeek (DLVO), extended DLVO, and other thermodynamic theories [118,121,123,124]. According to DLVO theory, the total energy between two approaching colloidal surfaces is the sum of the van der Waals attractive and electrostatic repulsive potentials. When two colloidal particles approach each other, van der Waals attractive forces increase, as do repulsive forces due to an overlap of the electrical double layers. A secondary attractive energy minimum responsible for weak adhesion is thus formed. At a closer approach, combining these forces produces a deep, attractive energy well, referred to as the primary minimum. Between the primary and secondary minima, an energy barrier slows the rate of particles falling into the primary minimum. Born repulsion forces dominate by overlapping electron shells at a closer distance than the primary energy minimum. Particles loosely trapped in the secondary minimum will only stay there if the energy barrier is sufficiently high to prevent them from falling into the primary minimum at a closer distance of approach [121,123,124]. It is important to note that adhesion due to electrostatic forces does not imply that surfaces are touching.

Reversible physical interactions can be dissociated by localized chemical environments and can be classified into nonspecific and specific interactions [125]. Nonspecific interactions include van der Waals attractive forces, hydrophobic association, and electrostatic interactions. Hydrophobic association is driven by an increase in water molecule entropy as highly ordered water structures surrounding approaching hydrophobic surfaces dissociate. Specific interactions require molecular functional groups. Nonspecific interactions allow bacterial cells to bind to surfaces, and specific chemical interactions become more significant at closer distances of approach [120].

Specific and stronger molecular interactions between bacterial and dairy food matrix surfaces that are nonreversible can occur. Adhesion mechanisms include interactions with bacterial structures, such as polysaccharides and lipoteichoic and teichoic acids [75,120,122]. Bacterial cells become more firmly attached to a surface, resulting in nonreversible adhesion due to the synthesis of extracellular adhesive materials. An example of bacterial adhesion is selective bridging by surface polymeric structures, such as capsules, fimbriae, and pili, and the presence of an extracellular slime layer consisting of glycoproteins, glycolipids, and exopolysaccharides [113,120,125]. At lower ionic strengths, the electrostatic energy barrier increases according to DLVO theory, and bacterial cells move further away from a charged surface, making it harder for EPS and nanofibers to extend from the bacterial cell to a dairy food matrix surface. This decreases the ability of bacterial cells to adhere to a surface, consistent with DLVO electrostatic theory [121,123,125].

#### 6.1.1. Bacterial Hydrophobicity

Bacterial surface hydrophobicity is one of the important bacterial factors affecting adhesion [126]. In general, bacteria with hydrophobic characteristics prefer to adhere to hydrophobic surfaces. The chemical functional groups on the surface of bacteria are primarily responsible for hydrophobicity. Bacterial surface hydrophobicity can be measured by contact angle measurements, such as the sessile drop method [127,128], partitioning of bacteria in a two-phase solvent system [129], hydrophobic interaction chromatography [130], fluorescent dye binding measurements, and bacterial adhesion to hexadecane, hydrocarbon, or polystyrene [131,132,133]. Bacterial surface differences in hydrophobicity are attributed to growth media, bacterial age, and surface structure [134].

Hydrophobic bacterial surfaces appear more prone to attach to a given substrate with similar hydrophobic characteristics than hydrophilic bacteria [127], all other types of interaction being equal. Although staphylococci generally exhibit minimal adhesion to more hydrophilic cellulose acetate, two *Staphylococcus epidermidis* strains, with more hydrophobic surface characteristics than *Staphylococcus saprophyticus*, demonstrated a much stronger adhesion to a hydrophobic poly(tetrafluoroethylene-co-hexafluoropropylene)-fluorocarbon (FEP) surface [135]. In this study, *S. epidermidis* treated with pepsin or extracted with aqueous phenol produced cells with decreased hydrophobicity and decreased adhesion to FEP [135]. The adherence of two *Streptococcus sanguis* strains and two *Streptococcus mutans* strains to surface-modified glass slides rendered with hydrophilic, ampholytic, or hydrophobic properties showed differences in adherence, depending on the physicochemical surface properties, with the more hydrophobic strains having greater adherence to hydrophobic glass slides [136].

#### 6.1.2. Milk Fat Globule Hydrophobicity

The MFGM exhibits hydrophobic and hydrophilic domains due to the diverse lipid and protein composition. The fatty acid alkyl chains of phospholipids, triglycerides, and cholesterol are responsible for the hydrophobic characteristics of the MFGM, whilst the polar head groups of phospholipids, sphingolipids, and glycoproteins are primarily responsible for the hydrophilic qualities. Transmembrane proteins tend to be hydrophobic and are in closer proximity to the hydrophobic alkyl chains of phospholipids. This amphipathic nature allows the MFGM to serve as an effective barrier, stabilizing the fat globule in an aqueous environment and enabling interactions with components in the surrounding aqueous phase, including probiotics. Hydrophilic surfaces are more resistant to bacterial adhesion than hydrophobic surfaces [136]. The hydrophobicity of MFGs can be measured by contact angles and by the fluorescence dye 8-anilino-1-naphthalene sulfonate (ANS) fluorescence probe method [121,123,129,137].

#### 6.1.3. Bacterial Surface Charge

Bacterial surface charge is another important determinant of bacterial attachment [118,138]. Most colloidal particles have an electric charge (more often negative than positive) and are dispersed as an aqueous suspension due to the ionization of the surface groups [122]. Surface electrostatic effects are quantified by the ζ-potential, which is the potential difference (in units of volts) between the hydrodynamic surface of shear and a point an infinite distance away from a particle. It must be noted that this is not a surface charge, which is an inherent property of a particle, and can vary considerably depending on the ionic strength of the suspending medium. Therefore, a suspending medium’s ionic conditions and pH must be precisely known in any reference to a ζ-potential. MFGs have a negative ζ-potential of around −10 mV at pH 6.7 and ionic strength of 80 mM [139,140]. The surface charge of microorganisms can be determined by isoelectric equilibrium analysis [141], electrostatic interaction chromatography [142], and colloidal titration [143].

A significantly higher absolute ζ-potential (negative) was observed for *L. acidophilus*, *L. casei*, *and L. delbrueckii* species, with higher adsorption to Caco-2/goblet cells in the presence of MFG phospholipids compared to controls without milk phospholipids [113]. In this study, *L. plantarum* had lower adsorption and did not show a significant difference in ζ-potential in the presence of milk phospholipids compared to a control. Bacterial surface charge is species-specific and depends on factors such as growth medium, pH, surface molecular architecture, and age of the bacteria.

#### 6.1.4. Milk Fat Globule Surface Roughness

Surface roughness is a three-dimensional parameter categorized into four groups: amplitude parameters, spacing parameters, functional parameters, and hybrid parameters according to the expected functionality of the surface [144]. Atomic force microscopy can characterize the surface topography and nanoscale features of biological materials, including milk fat globules. Rough surfaces are reported to harbor 25× more bacteria than smooth surfaces due to the greater surface area available for higher amounts of colonization [145,146].

The adherence of *P. aeruginosa*, *Ralstonia pickettii*, and *Staphylococcus epidermidis* to rougher surfaces was reported to be significantly higher than for smoother surfaces [147]. Higher surface roughness and lower ζ-potential were reported for globules in mastitic milk compared to fresh globules [148]; however, *L. fermentum* had a lower binding affinity to mastitic milk fat globules, likely because the reduction of specific surface proteins or alterations in the structure of glycoproteins in the mastitic MFGM may decrease the availability of attachment sites for the bacteria, resulting in reduced adhesion.

### 6.2. Chemical Mechanisms

Chemical adherence of bacterial cells to the MFGM involves surface proteins, including MUC 1, PAS6/7, butyrophilin, cluster of differentiation (CD 36), phospholipids, glycophospholipids, and glycosides [36,125,149]. However, specific components of the MFGM and the mechanisms by which they interact with probiotic bacteria have not yet been fully elucidated. The binding regions are currently speculative based on cell surface studies involving pathogenic bacteria [35,42]. Although there is no research specifically on the mechanism of LAB binding to MFGM-associated glycoproteins (such as MUC1), studies have reported that MFGM competes with epithelial cells to bind to pathogenic bacteria, thus minimizing adhesion to epithelial cells [42]. It is hypothesized that MUC1 may also influence the adherence of LAB to epithelial cells, although this remains untested [35].

The primary area where bacteria bind to surfaces is the cell surface calyx, which comprises two critical components: exopolysaccharides and surface proteins, which we will refer to as surface layer proteins (S-layer proteins) on the bacterial cell surface. S-layer proteins consist of identical subunits of one or, in a few cases, two proteins or glycoproteins. The amount of S-layer proteins differs between *Lactobacillus* strains, but when present, these are the most prevalent cellular proteins. Lactobacillus S-layer proteins have been isolated from porcine intestines and feces [150], from *L. helveticus* fb213, *L. acidophilus* fb116, and *L. acidophilus* fb214 [151], and *L. kefir* and *L. parakefir* [152]. In some strains of *Lactobacillus*, the absence of S-layer proteins reduces the capacity of bacterial cells to bind to surfaces, such as food and dairy matrices and mucosal and biomaterial surfaces [153]. A higher binding capacity of *Lactobacillus reuteri* to the MFGM was observed in the presence of S-layer proteins [131]. Furthermore, surface pili of LGG have a key adhesive role in bacterial adhesion to the MFGM [117].

MFGM components can be dislodged from the MFG surface in processed dairy products, for example, in the manufacture of cream where MFGM fragments partition into the buttermilk phase; these MFGM fragments could subsequently bind with probiotic microorganisms. Genes regulating S-layer protein components in LAB (NCFM *L. acidophilus*, 33199 *L. gallinarum*, 1063-S *L. reuteri*, and 53103 *L. rhamnosus*) were reported to be the major determinant in binding with intact and fragmented MFGM from raw and pasteurized creams, buttermilk, and buttermilk powder [154].

### 6.3. Environmental Factors

Environmental factors, such as temperature, pH, exposure time, bacterial concentration, and the presence of antibiotics will affect bacterial adhesion. The pH and the ionic strength of a suspending buffer affect bacteria adhesion by changing the charge and surface hydrophobicity of both bacterial and dairy food matrix surfaces. Protonation near the isoelectric point of proteins reduces the net charge and makes hydrophobic associations more pronounced. Extreme pH increases net charge and electrostatic repulsion [155]. The pH can modify LAB surface charge due to proteins, peptidoglycan, teichoic acid, and phospholipids [156].

The presence of antibiotics reduces bacterial adherence to surfaces, depending on the dose and bacterial susceptibility. Arciola al. reported less antibiotic sensitivity of attached *S. epidermidis* (bacterial biofilm) than non-adsorbing cells (planktonic) due to a protective biofilm matrix, reduced antibiotic penetration, altered gene expression, and adaptive resistance mechanisms that develop in the biofilm environment [157]. These authors proposed that adhesion may impact bacterial resistance to unfavorable environments (lower growth rate) by producing extracellular polymeric substances.

Bacterial adhesion can be affected by temperature. At higher temperatures, specific bacterial surface polymers, such as exopolysaccharides and proteins, can undergo conformational changes due to a reduction in viscosity. This loss of structural integrity can reduce the ability to bind effectively to surfaces, weakening bacterial adhesion. Additionally, studies have shown that certain bacteria have a larger surface area at lower temperatures (10 °C) compared to 35 °C [158] because, at lower temperatures, bacteria often respond by increasing the proportion of unsaturated fatty acids in the membranes which otherwise maintain membrane fluidity. This adaptation may increase bacterial surface area at lower temperatures (e.g., 10 °C), as observed by Herald and Zottola [158]. Increasing surface area could improve adhesion by enhancing contact points between the bacteria and the substrate. The enlarged surface area can also improve the ability of bacterial cells to absorb nutrients from the environment, enhance heat retention in colder conditions, and optimize the overall cellular function under thermal stress.

Temperature also impacts the quantity of flagella [159]. At lower temperatures, some flagellated bacteria can increase the number of flagella to enhance mobility and the ability to navigate in viscous or cold environments. This greater mobility can facilitate closer proximity of cells to the MFG, increasing the likelihood of surface interactions, assisting in surface exploration by the cells to determine the optimal area of interaction, and contributing to weak initial attachment, thus enabling bacteria to make brief contact with surfaces before more permanent adhesins, such as fimbriae or surface proteins, come into play. In contrast, these bacteria can decrease the number of flagella or modify their flagellar structure at higher temperatures, leading to more stable, less motile cells. A reduction in flagella could enhance adhesion by promoting a more sessile lifestyle, where bacteria prioritize attachment and colonization over motility.

## 7. Conclusions and Future Directions

This review offers a comprehensive analysis of current understanding of the composition and functionality of MFGs, probiotic bacteria, interactions, and the role of the MFGM in either promoting or inhibiting bacterial adhesion to the intestinal epithelium, as well as the effects these interactions have on the host. Recent research indicates that the MFGM can affect probiotic activity in the host by enhancing the viability of probiotics during transit through the gastrointestinal tract and promoting colonization, leading to beneficial health outcomes. These properties suggest that MFGM material holds significant potential as a carrier for probiotics to the intestine, contributing to positive health effects. However, significant gaps remain in understanding the precise molecular mechanisms underlying these interactions, particularly in relation to different probiotic strains and their growth, survival, and functionality in various dairy matrices.

Future research should explore how MFGM composition influences the species-specific interactions with probiotics and the impact of processing conditions on MFGM functionality. Additionally, more studies are needed to assess the long-term health effects of using MFGM as a probiotic carrier. Some suggestions for future research directions are as follows:

- Investigate the interactions of probiotic survival, growth, and functionality and how different probiotic strains interact with MFGM components.
- Explore ways to optimize MFGM components (e.g., phospholipids, sphingomyelin, cholesterol) to enhance probiotic efficacy.
- Consider individual factors such as age, gut microbiota, and diet to develop tailored probiotic interventions.
- Expand in vivo research on the long-term effects of MFGM–probiotic combinations on gut health and immune function.
- Investigate how MFGM–probiotic interactions can be used in functional foods and improve viability during storage and digestion.
- Explore the combined effects of MFGM and prebiotics to enhance probiotic efficacy.
- Further investigate the mechanisms of MFGM–probiotic interactions in the gastrointestinal tract and broader gut microbiome and host health.
- Assess the potential for incorporating MFGM–probiotic formulations into commercial food products.

## Figures and Tables

**Table 1:** Experimental evidence for health benefits of MFGM supplementation on human host health.

Growth, Development, and Metabolic Health	Gut Barrier Integrity and Gut Microbiota	Immunity	Cognitive Function (Mental Development)
Reduces susceptibility to obesity in adult life by preventing adipocyte hypertropia [47]. MFGM-enriched formulas meet the primary safety endpoint of non-inferiority in weight gain compared to the control formula [48]. Adequate growth throughout the first year of life [49]. Increases DHA availability which could be of importance for newborn growth and brain development [50]. Modifies the serum metabolome and reduces metabolic divergence [51]. In comparison to the breast-feeding group, the MFGM-enriched formula feeding group had similar development rates in terms of body weight, recumbent length, head circumference, and BMI [52]. Improves micronutrient status, energy metabolism, and growth [15].	Decreases episodes of fever, diarrhea, and constipation [53]. Fewer diarrheal episodes in infants of 6 to 11 months old who consumed complementary food with MFGM-enriched protein [54]. Higher probiotic colonization and lower pathogen colonization after treatment with MFGM [55]. Protects host against L. monocytogenes infection [56]. Similar intestinal development to when consuming mother’s milk, and promotes the development of intestinal microbiome and protects against inflammation [57].	Accelerates neurodevelopmental profile of infants [58]. Reduces the risk of AOM in infants and has immunomodulatory effects on humoral response against pneumococcus vaccine [59]. Cytokine profile of the MFGM group approaches that of breastfed infants [60]. Significantly fewer episodes of infection-related bloody diarrhea and improving metabolic regulation which may lead to enhanced immunity [15].	Improves neurocognitive development scores—cognitive, motor, and verbal scores [61]. Improves emotional and behavioral regulation in preschool children [53]. Accelerates neurodevelopment and attention scores in infants [58]. Reduces the gap in cognitive development between breastfed and formula-fed infants [61]. Improves social, emotional, short-term memory, and general adaptive behavior scores [62].

AOM: acute otitis media; BMI: body mass index; DHA: docosahexaenoic acid; MFGM: milk fat globule membrane.

**Table 2:** Clinical evidence of the main health benefits of probiotic dietary supplementation.

Gut Health and Gut Microbiota	Immune Modulation
Restores normal microbiota composition and function in antibiotic-treated and cesarean-born infants [79]. Induces colonization resistance and alleviates harmful effects of antibiotics on the gut microbiota and antibiotic resistome [80]. Early supplementation colonizes the preterm gut and affects potential pathogen colonization [81]. Allows targeted manipulation of the enteric microbiota and reduced abundance of bacterial taxa associated with the development of necrotizing enterocolitis [82]. Early administration to low-birth-weight infants is useful in promoting normal intestinal flora [83]. Effective in preventing antibiotic-associated diarrhea in children [84]. Helps to establish a healthy intestinal microbiota and improves intestinal barrier function [85]. Early administration improves infant gut health by reducing pathogen colonization [86]. Administration of a probiotic mixture reduces inconsolable crying in exclusively breastfed infants [87]. Reduces gastric distension, accelerates gastric emptying, and diminishes the frequency of regurgitation [88]. Efficacious in the treatment of acute diarrhea, reducing the frequency, duration, and recrudescence rate of the disease [89].	Used in the primary prevention of allergic diseases in neonates [90]. Exerts immunomodulatory effects, including enhanced production of intestinal secretory IgA [91]. Offers a safe and effective mode of promoting the immune protective potential of breast-feeding, and provides protection against atopic eczema during the first two years of life [92]. Maturation of the immune system in neonates by induction of Th1/Th2/Th3 response [93]. Reduces the severity of *E. coli* O157:H7 infection by enhanced humoral and cellular immune responses [94]. Reduction in the incidence of neonatal necrotizing enterocolitis by activation of the inflammatory cascade [95]. Induces an immunologic response by increasing the production of virus-neutralizing antibodies against poliomyelitis viruses [96]. Balances the Th1/Th2 immune response and antibody generation in healthy term infants after vaccination [85]. Helps to maintain fecal SIgA levels and stimulates the development of a mucosal immune response [97]. Enhances immunity by improved levels of fecal SIgA and T-cell subsets in peripheral blood [98].

IgA: immunoglobulin A; SIgA; secretory immunoglobulin A; Th: T helper cells.

**Table 3:** Studies on the impact of probiotics and MFGM interactions.

Supplementation	Method	Effect	References
MFGM-derived MPL + *P. acidilactici* OSU-PECh-L, *P. acidilactici* OSU-PECh-3A, *L. plantarum* OSU-PECh-BB, L. reuteri OSUPECh-48, *L. casei* OSU-PECh-C, *L. paracasei* OSU-PECh-BA, or *L. paracasei* OSU-PECh-3B	Bacteria grown in media supplemented with 0.5% milk MPL for 8 to 9 h at 37 °C. Bacteria were added to a Caco-2 monolayer and incubated for 3 h at 37 °C. Caco-2 cells were washed, bacterial adherence measured.	MPL supplementation showed three out of seven strains with increased adhesion to intestinal cells compared to the control without MPL.	[114]
Whey-derived MFGM-10 + LGG	Six-week-old BALB/c male mice were gavaged with MFGM-10 (5 g/L) and LGG for three days, and cecum and fecal LGG cell counts in mice were analyzed.	LGG viability was increased after GI passage in the treatments combining LGG and MFGM-10 compared to the MFGM-10 or probiotic treatments alone.	[16]
MFGM-derived MPL + *L. casei*, *L. delbrueckii*	Bacteria were cultured in a defined liquid medium enriched with 0.5% MPL for 17 h at 37 °C, added to Caco-2 and HT-29 goblet cells, and incubated for 2 h at 37 °C.	MPL-supplemented media increased the adhesion of *L. casei* and *L. delbrueckii* to Caco-2/goblet cells with increased surface of shear ζ-potential compared to the control without MPL.	[113]
MFGM-derived MPL concentrate + *Lactobacillus delbrueckii* subsp. bulgaricus 2038, *Streptococcus thermophilus* 1131	Male Sprague–Dawley rats were orally administrated with sphingomyelin/MPL concentrate alone or sphingomyelin/MPL with fermented milk containing *Lactobacillus delbrueckii* subsp. *bulgaricus* 2038, *Streptococcus thermophilus* 113.	Co-ingestion of sphingomyelin/MPL and fermented milk increased the absorption of dietary sphingomyelin approximately two-fold compared to sphingomyelin/MPL concentrate alone.	[115]
MFGM fractions + *Bifidobacterium lactis* NCC2818	NF-ĸB Reporter assay was carried out using an HT29C134 cell line and treated with an MFGM–probiotic combination, or MFGM or probiotic alone. B- and T-cell stimulation assays were carried out using 6–8-week-old C57BL/6 mice lymphocyte cells. IgA-secreting cell numbers in Peyer’s patches cell suspensions were evaluated after treatment with an MFGM–probiotic combination, or with MFGM or probiotic treatments alone.	MFGM and probiotic combination decreased NF-ĸB activation compared to these treatments separately. A significant increase in the number of intestinal IgA-secreting cells in the MFGM and probiotic treatment groups was observed.	[12]
MFG fraction + *Lactobacillus delbrueckii* ATCC 11842, *Bifidobacterium infantis* ATCC 15697, *Bifidobacterium longum* CGMCC 1.3006, *Lactobacillus acidophilus* CICC 6081, *Salmonella enterica* ATCC 13076, *Cronobacter sakazakii* ATCC 29544, and *Escherichia coli* CTCC 10665	Bacteria were co-cultured for 3 h at 37 °C in HT 29 cells which were pre-treated with human/caprine/bovine MFGs for 3 h at 37 °C. The lysate was collected and cultured in MRS media, and colony-forming units were counted in MRS after 18 h of incubation at 37 °C.	All types of MFGs significantly enhanced probiotic adhesion compared to the control group. Pathogen colonization ability was significantly reduced in MFG treatment groups compared to the control without the MFG fraction.	[55]
MFGM + *B. lactis* CNCM I-3446	For four weeks, freshly weaned mice received a daily dosage of MFGM and/or a probiotic, or a placebo. ELISPOT was used to count the number of mucosal IgA-secreting cells at the end of the supplementation period and 12 weeks later.	Combined probiotics and MFGM had the highest mucosal B- and T-cell proliferative response, showing a greater impact than either probiotic or MFGM treatments alone.	[116]
MFGM + *L. rhamnosus* (LGG)	LGG wild type and surface mutants (pili-depleted and EPS-deficient LGG) were exposed to 5 mg/mL MFGM extract for 1 h at 37 °C, applied to Caco-2 TC7 cells, and incubated for 2 h at 37 °C.	The presence of MFGM decreased the adhesion to host intestinal epithelial cells by blocking the pili adhesive sites of LGG.	[117]
MFGM-derived MPL + *Lactiplantibacillus plantarum*	Bacteria were cultured in a defined liquid medium enriched with 0.5% of MPL for 17 h at 37 °C and added to Caco-2 and HT-29 goblet cells and incubated for 2 h at 37 °C.	MPL-treated *L. plantarum* showed lower adhesion compared to a control without MPL.	[113]
MPL-rich milk protein, whey-derived MFGM-10, buttermilk + *Bifidobacterium longum* subsp. *infantis* ATCC 15697	Bifidobacteria were exposed to milk-derived powder for 1 h at 37 °C, and the bacteria were applied to HT-29 cells and incubated for 2 h at 37 °C.	MPL-rich milk protein and buttermilk decreased the adhesion of Bifidobacteria, whereas MFGM-10 did not alter the adhesion of ATCC 15697.	[22]

Caco-2/TC7 human colorectal adenocarcinoma cells; ELISPOT: enzyme-linked immune spot assay; EPS: exopolysaccharide; GI: gastrointestinal; HT29: human colon adenocarcinoma cell; IgA: immunoglobulin A; LGG: *L. rhamnosus* GG; MFGs: milk fat globules; MFGM: milk fat globule membrane; MFGM-10: Lacprodan^®^ (Arla Foods Ingredients, Aarhus, Denmark); MPL: milk phospholipid; MRS: De Man–Rogosa–Sharpe; NF-ĸB: nuclear factor kappa-light-chain-enhancer of activated B cells.

## Data Availability

Data are contained within the article.
